# Social support and post-traumatic stress symptoms: Longitudinal bidirectional relationships in the AURORA study

**DOI:** 10.1016/j.xjmad.2026.100180

**Published:** 2026-05-14

**Authors:** Justin LC. Santos, Sanne JH. van Rooij, Stacey L. House, Francesca L. Beaudoin, Xinming An, Thomas C. Neylan, Gari D. Clifford, Tanja Jovanovic, Sarah D. Linnstaedt, Scott L. Rauch, John P. Haran, Alan B. Storrow, Christopher Lewandowski, Paul I. Musey, Phyllis L. Hendry, Sophia Sheikh, Christopher W. Jones, Brittany E. Punches, Robert A. Swor, Lauren A. Hudak, Jose L. Pascual, Mark J. Seamon, Erica Harris, Claire Pearson, David A. Peak, Robert M. Domeier, Niels K. Rathlev, Brian J. O'Neil, Paulina Sergot, Leon D. Sanchez, Steven E. Bruce, Steven E. Harte, Ronald C. Kessler, Karestan C. Koenen, Kerry J. Ressler, Samuel A. McLean, Jennifer S. Stevens

**Affiliations:** a Department of Psychiatry and Behavioral Sciences, Emory University School of Medicine, Atlanta, GA 30332, USA; b Department of Psychiatry and Behavioral Sciences, Emory University School of Medicine, Atlanta, GA 30329, USA; c Department of Emergency Medicine, Washington University School of Medicine, St. Louis, MO 63110, USA; d Department of Epidemiology, Brown University, Providence, RI 02930, USA; e Department of Emergency Medicine, Brown University, Providence, RI 02930, USA; f Institute for Trauma Recovery, University of North Carolina at Chapel Hill, Chapel Hill, NC 27559, USA; g Department of Anesthesiology, University of North Carolina at Chapel Hill, Chapel Hill, NC 27559, USA; h Departments of Psychiatry and Neurology, University of California San Francisco, San Francisco, CA, 94143, USA; i Department of Biomedical Informatics, Emory University School of Medicine, Atlanta, GA 30332, USA; j Department of Biomedical Engineering, Georgia Institute of Technology and Emory University, Atlanta, GA 30332, USA; k Department of Psychiatry and Behavioral Neurosciences, Wayne State University, Detroit, MI 48202, USA; l Institute for Technology in Psychiatry, McLean Hospital, Belmont, MA, 02478, USA; m Department of Psychiatry, McLean Hospital, Belmont, MA 02478, USA; n Department of Psychiatry, Harvard Medical School, Boston, MA 02115, USA; o Department of Emergency Medicine, University of Massachusetts Chan Medical School, Worcester, MA 01655, USA; p Department of Emergency Medicine, Vanderbilt University Medical Center, Nashville, TN 37232, USA; q Department of Emergency Medicine, Henry Ford Health System, Detroit, MI 48202, USA; r Department of Emergency Medicine, Indiana University School of Medicine, Indianapolis, IN 46202, USA; s Department of Emergency Medicine, University of Florida College of Medicine -Jacksonville, Jacksonville, FL 32209, USA; t Department of Emergency Medicine, Cooper Medical School of Rowan University, Camden, NJ 08103, USA; u Department of Emergency Medicine, Ohio State University College of Medicine, Columbus, OH 43210, USA; v Ohio State University College of Nursing, Columbus, OH, 43210, USA; w Department of Emergency Medicine, Oakland University William Beaumont School of Medicine, Rochester, MI 48309, USA; x Department of Emergency Medicine, Emory University School of Medicine, Atlanta, GA 30329, USA; y Department of Surgery, Department of Neurosurgery, University of Pennsylvania, Philadelphia, PA 19104, USA; z Perelman School of Medicine, University of Pennsylvania, Philadelphia, PA 19104, USA; aa Department of Surgery, Division of Traumatology, Surgical Critical Care and Emergency Surgery, University of Pennsylvania, Philadelphia, PA 19104, USA; ab Department of Emergency Medicine, Einstein Medical Center, Philadelphia, PA 19107, USA; ac Department of Emergency Medicine, Wayne State University, Henry Ford St. John Hospital, Detroit, MI 48236, USA; ad Department of Emergency Medicine, Massachusetts General Hospital, Boston, MA 02114, USA; ae Department of Emergency Medicine, Harvard Medical School, Boston, MA 02115, USA; af Department of Emergency Medicine, Trinity Health-Ann Arbor, Ypsilanti, MI 48197, USA; ag Department of Emergency Medicine, University of Massachusetts Medical School-Baystate, Springfield, MA 01107, USA; ah Department of Emergency Medicine, Wayne State University, Detroit Receiving Hospital, Detroit, MI 48202, USA; ai Department of Emergency Medicine, McGovern Medical School at UTHealth, Houston, TX 77030, USA; aj Department of Emergency Medicine, Brigham and Women's Hospital, Boston, MA 02115, USA; ak Department of Psychological Sciences, University of Missouri, St. Louis, MO 63121, USA; al Department of Anesthesiology, University of Michigan Medical School, Ann Arbor, MI 48109, USA; am Department of Internal Medicine-Rheumatology, University of Michigan Medical School, Ann Arbor, MI 48109, USA; an Department of Health Care Policy, Harvard Medical School, Boston, MA 02115, USA; ao Department of Epidemiology, Harvard T.H. Chan School of Public Health, Harvard University, Boston, MA 02115, USA; ap Division of Depression and Anxiety, McLean Hospital, Belmont, MA 02478, USA; aq Department of Emergency Medicine, University of North Carolina at Chapel Hill, Chapel Hill, NC 27559, USA; ar Department of Psychiatry, University of North Carolina at Chapel Hill, Chapel Hill, NC 27559, USA

**Keywords:** PTSD, Cross-lagged Panel Model, Social Support

## Abstract

**Background:**

While social impairment is a hallmark of increasing severity of post-traumatic stress disorder (PTSD), the dynamics between social support and trauma recovery are less clear. Current theories suggest either social support is responsible for reducing PTSD severity (*social causation*), or worsening PTSD ultimately degrades supportive networks (*social erosion*). Furthermore, there is a paucity of research on social support and longitudinal trauma recovery within the civilian population. We hypothesized a bidirectional relationship exists between social support and PTSD, such that both social causation and erosion impact the trajectory of recovery at different times post-trauma.

**Methods:**

A total of n = 2943 participants were recruited following a traumatic event leading to Emergency Department visit in the AURORA study, reported perceived emotional support (PROMIS) and severity of PTSD symptoms (PCL-5) at 2 weeks, 8 weeks, 3 months, 6 months, and 12 months post trauma. PTSD symptom severity overall and within symptom criteria (B-E) were assessed.

**Results:**

A 5-wave cross-lagged panel model showed a statistically significant negative association between emotional support and subsequent PTSD symptoms across the majority of timepoints in the year following trauma. Concurrently, we observed a negative association between PTSD severity and subsequent perceived emotional support. Additional analyses showed emotional support was mainly correlated with subsequent negative mood and feelings, whereas avoidant behavior was most correlated with lower emotional support.

**Conclusion:**

Our analyses emphasize the importance of initiating social interventions and establishing robust social networks immediately after a traumatic event.

## 1 Introduction

Among the various presentations of post-traumatic stress disorder (PTSD), a shared aspect of patient experiences involves some form of social impairment. Independent of trauma type or patient demographic, compromised interpersonal connections are commonly observed [1], [2]. Epidemiological studies suggest an absence of pre-trauma social support is a significant risk factor for developing PTSD following a traumatic event [3], [4], [5]. Furthermore, post-trauma social support has been demonstrated to aid in recovery [6], [7]. However, PTSD symptoms also strain interpersonal relationships [5]. Longitudinal studies in civilian and military populations suggest social isolation and feelings of loneliness are also directly related to increased PTSD severity [8], [9]. While early social support can be protective of PTSD, this effect is potentially blunted in trauma survivors with maladapted social behavior. In this current study we aim to address the question surrounding directionality in the associations between social support and PTSD.

When considering directionality, there are currently two theories surrounding the causal links between social support and PTSD, namely social causation and social erosion [10], [11]. In social causation, robust and early support corresponds to a reduction of PTSD symptom severity. This is conceptually analogous to the idea of social buffering, in which a subject’s response to stressful stimuli is reduced in the presence of an affiliative organism [12]. This stress reduction has been observed in multiple animal models, from triads of guinea pigs, partnered rats, and mother-infant rodent dyads [13], [14], [15]. A similar effect was also replicated in both healthy, and trauma-exposed human populations [7], [16], [17]. Additionally studies in both civilian and military populations suggest that social support not only reduces stress but promotes resilience and may assist with treatment adherence, predicting more positive outcomes [18].

In contrast, social erosion suggests that PTSD symptoms are more responsible for undermining support and undermining interpersonal relationships. A longitudinal, prospective study of Gulf War veterans found that PTSD symptoms accounted for a later reduction in perceived social support ultimately degrading existing support networks [19]. Similarly, in another military study, Iraq war veterans were examined for the quality of their social networks, finding veterans with more severe PTSD symptoms were endorsing worsened interpersonal relationships within 6–9 months following a return from deployment [20]. Within the civilian context, there is still a need for further research. However, one such prospective study followed survivors of a traumatic event, from hospital admission through 6 years after, observing greater PTSD severity predicting negative perceptions of social support [21]. To some degree this is demonstrated in youth and adolescent populations, where increased trauma burden was associated with lower perceived social support. While this study did not observe trauma exposure reducing social support long term within their time frame, the team noted how longer studies may show significant social erosion [22]. Another study following individuals recovering from traumatic physical injury also found increased PTSD severity correlated with negatively perceived social support at one year post-trauma [23]. Consequently, avoidance-based motivation to self-isolate becomes reinforced, with degrading social connections making it difficult to implement and maintain necessary interventions and contributing to poorer patient outcomes.

Realistically, it is more likely that both social causation and social erosion act in concert to compound the severity of PTSD symptoms. Some studies observed a reciprocal association between social support and post-traumatic stress symptoms at different points within the same recovery time frame. A longitudinal study following a civilian population that experienced a natural disaster found that social support was protective of acute post-traumatic stress symptoms, but later in recovery, higher PTSD symptoms played a greater role in the reduction of perceived social support [10]. In another study, 9/11 survivors with higher PTSD symptoms perceived less support early on whereas those who maintained higher social support scores across the recovery time frame coincided with decreased PTSD symptoms [24]. Similarly, adult survivors of childhood sexual trauma showed longitudinally reciprocal associations between perceived social support and post-traumatic stress symptoms [25]. In another study, veterans endorsed social support mediating post-traumatic stress, while concurrently those with poorer mental health at baseline, struggled to establish and maintain social and interpersonal connections [26]. Regardless of the debate in directionality between social support and PTSD, the literature suggests that the difference in recovery is not between the overarching impact of social causation versus erosion, but in when post-trauma those concepts are most dominant. However collectively, the body of research on social support and PTSD is limited by either a narrow focus on specific traumatic events, loss to follow-up in later time points, or participant populations often recruited years after PTSD diagnosis [27], [28]. There are few studies in the acute post-trauma timeframe with large, representative samples.

The complexity of the association between social support and PTSD extends to symptom subtypes. The severity of avoidance and, to a lesser degree, hyperarousal symptoms, are thought to be primarily responsible for PTSD severity, and have been the subject of targeted social support studies. For example, we see a mirrored relationship between avoidant symptoms and social support, in which patients who isolate themselves degrade existing interpersonal relationships and exhibit an impairment of social functioning, leading to even poorer mental health symptoms later on [29]. Conversely, in a study analyzing 6-year outcomes following a mass casualty incident, higher social support was negatively correlated with post-traumatic avoidance symptoms [30]. More recently, a study on emotional avoidance following traumatic exposure found that individuals with high and early levels of perceived social support experienced a reduction in avoidance symptoms in the year following treatment [31]. A recent study on pediatric cancer patients showed that social support was associated with lower hyperarousal symptoms [32]. Altogether, this suggests that early social support may be protective of PTSD, specifically by mitigating avoidance and hyperarousal symptoms.

Building on these ideas, we investigated the bidirectional relationship between social support and PTSD, utilizing data from the Advancing Understanding of RecOvery after traumA (AURORA) study, a longitudinal, multi-site study focusing on neuropsychiatric outcomes in post-traumatic recovery [33]. Importantly, the AURORA study features dense sampling in the acute, post-trauma timeframe, on individuals presenting to the emergency department following various traumatic events. At regular intervals post-trauma (2 weeks, 8 weeks, 3 months, 6 months, and 12 months), participants reported on their perceptions of social support along with the intensity of PTSD symptoms at each timepoint. We hypothesized that, early after trauma, social support would be associated with lower subsequent acute post-traumatic stress symptoms. However, after several months of impairing stress-related symptoms, we predicted that post-traumatic stress would conversely be associated with declining social support. Finally, we were interested in exploring the effects of social support on various PTSD symptom clusters. We predicted that social support would correlate with reduced PTSD severity, primarily for avoidance and hyperarousal symptoms.

## 2 Materials and methods

### 2.1 Study population

These analyses utilize data from the longitudinal, prospective AURORA study. Participants were recruited from various emergency departments (EDs) across the United States, with n = 2943 individuals contributing psychosocial information at various timepoints over the course of recovery post-trauma. From demographic data to traumatic event questionnaires, and psychiatric self-reported measures, data was collected at 5 timepoints post-trauma, namely 2-weeks, 8-weeks, 3-months, 6-months, and 12-months, with individuals reporting from September 2017 through June 2020.

Importantly, this study aimed to represent a diverse collection of inciting criteria A traumatic events (TEs). TEs include physical and sexual assault, falls > 10 feet, motor vehicle collisions, and any other event in which the individuals were directly exposed or witnessed serious injury, violence, or death. Additionally, all exposures were validated by research assistants. Medical exclusion criteria include administration of general anesthesia, active opioid use, long bone fractures, hemorrhagic injury, solid organ injury, pregnancy, active breastfeeding, self-inflicted or occupational injury, and visual or auditory impairment. Additional exclusion criteria include poor fluency in the English language, ongoing domestic violence, and imprisonment. Final demographics are listed in Table 1.

**Table 1:** Demographics of Final Sample (n = 2943).

Variable	Count (%)/Mean (SD)
*Gender*	
Sex(M)	1124 (38%)
Sex(F)	1818 (64%)
No Response	1
*Age*	35.90 (13.29)
*Race/Ethnicity*	
Hispanic/Latin American	342 (12%)
White-American	1020 (35%)
Black-American	1458 (50%)
"Other" American	111 (3%)
*Employment*	
Employed	1902 (65%)
Retired	66 (2%)
Homemaker	56 (2%)
Student	98 (3%)
Unemployed, disabled, or other	473 (16%)
No Response	348 (12%)
*Income (Total Family Income)*	
< $19,000	850 (29%)
$19,001-$35,000	794 (27%)
$35,001-$50,000	353 (12%)
$50,001-$75,000	216 (7%)
$75,001-$100,000	173 (6%)
> $100,000	195 (7%)
No Response	362 (12%)
*% with probable PTSD (PCL-5 >31)*	
Week 2 (acute stress disorder)	43.2%
Week 8	36.7%
Month 3	29.7%
Month 6	22.6%
Month 12	16.1%
*Trauma Type*	
Motor Vehicle Collision	2194 (74.5%)
Physical Assault	271 (9.2%)
Sexual Assault	17 (0.6%)
Fall	213 (7.2%)
Mass Trauma Incident	12 (0.4%)
Non-motorized Collision	53 (1.8%)
Poisoning	2 (0.1%)
Burns	14 (0.5%)
Animal-related	63 (2.1%)
Other	104 (3.5%)

### 2.2 Social support

For the evaluation of social support, participants were scored on a subset of the PROMIS Emotional Support questionnaire – Short Form 4a with modified question stems and responses. Altogether, the NIH PROMIS measures are widely utilized and show high inter-rater reliability in measuring social health [34]. Specifically, the emotional support subset demonstrates robust reliability; Cronbach’s alpha = 0.97 [35]. The AURORA study summed 3 items which evaluated the quality of perceived support, self-worth, and interpersonal relationships, to create an ‘emotional support score’ ranging from 3 to 15. On a scale of 1 (rarely) to 5 (very often), participants endorsed within their relationships, how well they felt listened to, were made to feel appreciated, or connected with them on a ‘bad day’, throughout the past month. This emotional support score was collected at all 5 timepoints listed above and demonstrates strong internal reliability (Cronbach’s 2-week α = 0.88, Cronbach’s 6-month α = 0.92).

### 2.3 Symptoms of post-traumatic stress disorder

PTSD was assessed at 2-weeks, 8-weeks, 3-months, 6-months, and 12-months post trauma, through the PTSD Checklist for Diagnostic and Statistical Manual for Mental Disorders – 5th Edition (DSM-5), a 20 item self-reported questionnaire rating the severity of post-trauma stress symptoms. On a scale of 0 (not at all) to 4 (extremely), survey questions evaluated a variety of symptoms including reexperiencing, hyperarousal and reactivity, trauma-related avoidance, and negative changes in mood and cognition, correlating with various PTSD sub-criteria. The 20 items are summated for a total score ranging from 0 to 80, with sub scores representing subtotals of different survey criteria.

### 2.4 Statistical analysis

Regarding our primary dataset, preliminary little’s MCAR test suggested that data was not completely missing at random (p-value = 1.41 ×10^13^). However, subsequent regression analyses demonstrate that missingness at the 8-week time point was associated with baseline emotional support while missingness at later time points were not associated with observed variables. This suggests that our data may be more consistent with a missing at random assumption, and appropriate for multiple imputation with the *aregImpute* function in the *Hmisc* R package, best suited for clinical and psychosocial data types. This method employs 5 imputations using PROMIS emotional support scores, and PCL-5 scores at the 2-week, 8-week, 3-month, 6-month, and 12-month time points to generate robust estimates that predict the missing data. The number of missing participants per time point is as follows: 2-week: 325, 8-week: 456, 3-month: 612, 6-month: 985, 12-month: 1403. To test our concurrent hypotheses that emotional support would predict reduced longitudinal PTSD symptoms, and PTSD symptoms would be associated with lesser subsequent emotional support, we conducted a 5-wave, longitudinal, cross-lagged panel model using the *lavaan* package in R version 4.3.2. Each wave corresponded to a post-trauma timepoint (2-weeks, 8-weeks, 3-months, 6-months), with beta-value coefficients that quantify associations between emotional support and subsequent PTSD scores, along with the reciprocal relationship of PTSD symptom severity and later emotional support.

We then tested the hypothesis that emotional support would primarily show negative associations with later avoidance and hyperarousal symptoms. Based on statistically significant lagged pathways in the cross-lagged model, we conducted additional regression models, exploring how emotional support would predict PTSD symptom clusters (B, C, D, and E). For the reciprocal direction, we sought to determine which symptom clusters were most dominant in predicting later worsened social support. We conducted FDR p-value corrections for the models investigating how social support predicts symptom clusters. We also employed a Steiger’s test, optimal for dependent correlations, to determine if the differences in our regression models were statistically significant [36].

## 3 Results

**Fig. 1:**
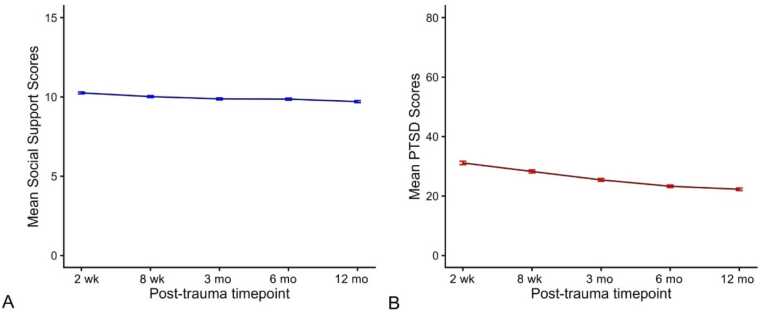
Longitudinal progression of mean social support scores and mean PTSD symptom scores across 5 timepoints post-trauma (2-weeks, 8 weeks, 3 months, 6 months, 12 months). Error bars indicate + /- 1 SE.

The longitudinal trend of PROMIS emotional support scores is displayed in Fig. 1**A**. The longitudinal trend of PCL-5 scores is displayed in Fig. 1**B.** Consistent with our expectations, traumatic stress symptoms decreased over the course of recovery. However, interestingly, we also observed a minor reduction in support over time as well.

### 3.1 Cross-lagged effects between emotional support and PTSD scores

**Fig. 2:**
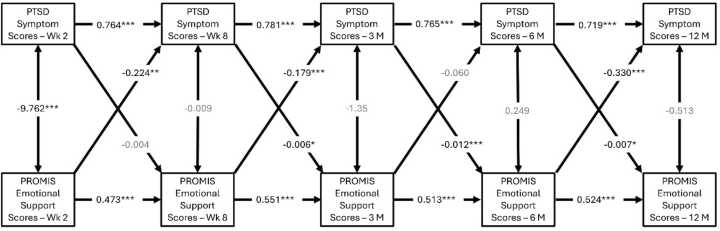

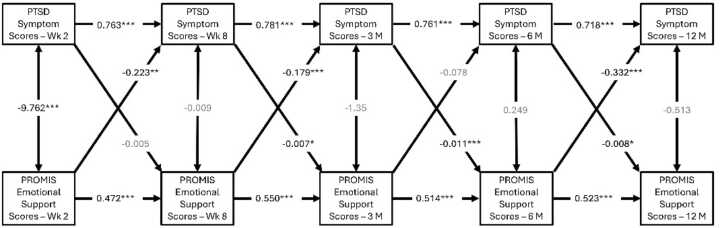

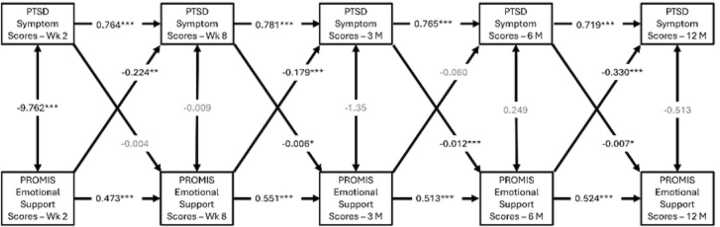
**A-C**: Cross-lagged panel model showing reciprocal relationships between emotional support and PTSD symptom scores. Emotional support is negatively correlated with PTSD scores at 8 weeks, 3 months, and 12 months post-trauma, while PTSD symptoms predicted worsened emotional support at 3 months, 6 months, 12 months post-trauma. (B values reported; p values <0.001***, <0.01**, <0.05**). Models B and C controlled for sex and trauma type respectively.

Emotional support negatively predicted subsequent PTSD symptom severity at 8 weeks (β=-0.038, p = 0.002), 3-months (β=-0.031, p = 0.005), and 12-months (β=-0.061, p < 0.001), respectively (Fig. 2). Translating this effect, for each unit increase of emotional support, the model predicted an approximate subsequent reduction of PTSD symptom scores by 1.49 units at 8-weeks, 1.19 units at 3-months, and 2.2 units at 12-months post trauma. Given that the PCL-5 ranges from 0 to 80, a 2.2-point reduction reflects at least a 2.75% decrease in symptoms.

Conversely, higher PTSD symptom severity at the 8-week, 3-month, and 6-month time points were negatively correlated with subsequent emotional support scores at 3 months (β=-0.032, p = 0.037), 6 months (β=-0.062, p < 0.001), and 12 months (β=-0.036, p = 0.023), post-trauma (Fig. 2**A**). Additional cross-lagged models controlling for sex (Fig. 2**B**) and trauma-type (Fig. 2**C**) showed similar results.

### 3.2 Emotional support and PTSD symptom clusters

**Fig. 3:**
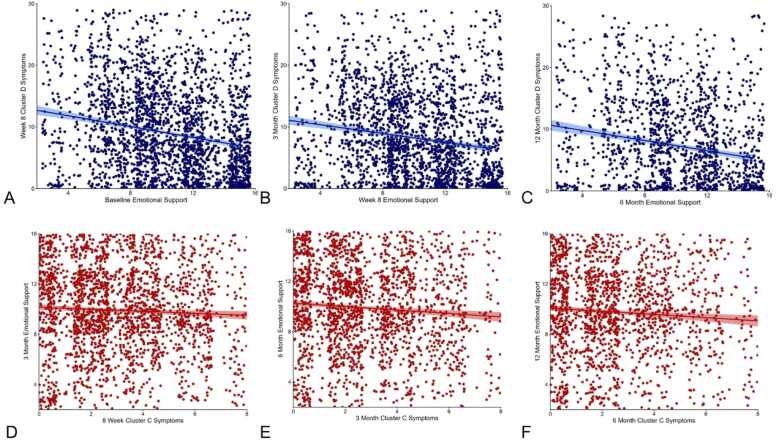
These plots highlight the relationships between emotional support and PTSD symptom subtypes. Emotional support predicted fewer Cluster D (y-axis 0–28 points) symptoms at the 8-week, 3-month, and 12-month time points (A-C), while Cluster C symptoms (x-axis 0–8 points) predicted lower emotional support scores at the 3-month, 6-month, 12-month time points (D-F).

We then explored the magnitude of effects that various PTSD symptom subtypes were contributing to the significant lagged associations. The relationship between emotional support and subsequent PTSD symptom severity within each symptom type is shown in Table 2**.** Emotional support was most strongly correlated with subsequent Cluster D symptoms, at the 8-week (β=-0.426, p < 0.001, df=2570), 3-month (β=-0.294, p < 0.001, df=2395), and 12-month (β=-0.389, p < 0.001, df=1587) time points (Fig. 3**A-C,** Table 2). Support also negatively correlated with the other PTSD symptom clusters, including reexperiencing symptoms, avoidant behavior, and hyperarousal and reactivity. A comprehensive display of the relationship between emotional support and PTSD symptom types is shown in Table 2**.** A subsequent Steiger’s test of the correlations in our regression models shows statistically significant differences between emotional support and PTSD symptom scores.

**Table 2:** Associations between emotional support and lagged PTSD symptom severity by cluster.

Dependent variable (PTSD symptom severity)	Beta	SE	t	p	FDR-corrected p
*2-week emotional support predicting 8-week PTSD symptoms*					
Cluster B	-0.173	0.033	-5.222	< 0.001	< 0.001
Cluster C	-0.059	0.146	-4.085	< 0.001	< 0.001
Cluster D	-0.426	0.045	-9.480	< 0.001	< 0.001
Cluster E	-0.287	0.409	-7.031	< 0.001	< 0.001
*8-week emotional support predicting 3-month PTSD symptoms*					
Cluster B	-0.080	0.035	-2.267	0.024	0.031
Cluster C	-0.029	0.016	-1.869	0.062	0.062
Cluster D	-0.294	0.047	-6.195	< 0.001	< 0.001
Cluster E	-0.174	0.042	-4.189	< 0.001	< 0.001
*6-month emotional support predicting 12-month PTSD symptoms*					
Cluster B	-0.214	0.038	-5.591	< 0.001	< 0.001
Cluster C	-0.073	0.017	-4.384	< 0.001	< 0.001
Cluster D	-0.389	0.054	-7.265	< 0.001	< 0.001
Cluster E	-0.248	0.046	-5.441	< 0.001	< 0.001

In the opposite direction, associations between various PTSD symptom clusters and subsequent emotional support are displayed in Table 3. We discovered that Cluster C symptoms, appeared to be most strongly correlated with subsequent emotional support scores at 3 months (β=-0.066, p = 0.016, df=2570), 6 months (β=-0.080, p = 0.004, df=2395), and 12 months (β=-0.054, p < 0.001, df=2029) post-trauma (Fig. 3**D-F,** Table 3). However, a Steiger’s test did not detect a significant difference between these models. Overall, increasing PTSD symptom clusters, including reexperiencing, avoidance, hyperarousal and reactivity negatively correlate with emotional support across all time points.

**Table 3:** Associations between PTSD symptom severity (by cluster) and lagged emotional support.

Independent variable (PTSD symptoms)	Beta	SE	t	p values	FDR-corrected p value
*8-week PTSD symptoms predicting 3-month emotional support*					
Cluster B	-0.025	0.012	-2.102	0.036	0.036
Cluster C	-0.066	0.027	-2.419	0.016	0.021
Cluster D	-0.048	0.009	-5.501	< 0.001	< 0.001
Cluster E	-0.025	0.010	-2.622	0.009	0.018
*3-month PTSD symptoms predicting 6-month emotional support*					
Cluster B	-0.044	0.012	-3.581	< 0.001	< 0.001
Cluster C	-0.080	0.028	-2.916	0.004	0.004
Cluster D	-0.061	0.009	-6.810	< 0.001	< 0.001
Cluster E	-0.050	0.010	-4.843	< 0.001	< 0.001
*6-month PTSD symptoms predicting 12-month emotional support*					
Cluster B	-0.046	0.014	-3.337	< 0.001	0.001
Cluster C	-0.081	0.033	-2.48	0.013	0.013
Cluster D	-0.054	0.011	-5.171	< 0.001	< 0.001
Cluster E	-0.055	0.012	-4.507	< 0.001	< 0.001

## 4 Discussion

Our current study explores the reciprocal associations of social support on PTSD symptom severity. Consistent with our hypothesis, both emotional support and PTSD symptoms exert bidirectional effects across timepoints post-trauma. Emotional support was significantly associated with reductions in post-trauma stress throughout the first-year post-trauma. Conversely, PTSD symptoms were correlated with lower support scores beginning at the 8-week time point. Emotional support is also associated with a global reduction of PTSD symptom clusters suggesting that support can promote resilience regardless of the inciting traumatic event or unique constellation of symptoms. Importantly, these results demonstrate how acute post-trauma social support relates to longitudinal patient outcomes.

Like previous cross-lagged studies, we found that both social causation and social erosion play a role in post-trauma recovery [10]. While prior studies provide rich longitudinal investigation of the relationships between PTSD and the social environment in the years and decades following trauma exposure, our findings provide novel insight into the initial emergence of traumatic stress symptoms and early recovery. For example, a 4-wave cross-lagged study observed 9/11 survivors over the course of 14 years, beginning with social support assessment in time points nearly a decade after the inciting traumatic event [24]. While they were able to monitor the dynamics of PTSD symptoms across multiple decades, conclusions on lagged effects involving social support were limited by delayed data collection. Still, they observed that early in recovery, PTSD reduced social support, while later social support buffered against PTSD symptoms. Similarly, among survivors of a mass casualty event, participants were recruited nearly a year following the precipitating traumatic event [37]. Surprisingly, they found that neither social support nor PTSD seemed to significantly influence one another over the course of the study. The longitudinal study that focused earliest on the post-trauma recovery period was conducted among survivors of a natural disaster in Mexico, assessed from 6 to 24 months post trauma. Here, social support was only dominant in the early points, 6- and 12-months post-trauma, and PTSD symptoms contributed to social degradation beginning at 18 months post-trauma [10].

PTSD can be formally diagnosed at 1-month post-trauma so this is an important threshold to evaluate the quality of an individual’s social support. Moreover, social networks on a longer time frame may evolve so differently, complicating eventual conclusions. These factors motivated our focus on the early weeks and months following a traumatic event. We found that the reciprocal effects of emotional support and PTSD were imbalanced, such that social support negatively predicted post-traumatic stress severity across the entire length of the study, while PTSD symptoms were associated with lower perceived emotional support at the 3 month and later time points. The findings support the hypothesis that immediate support in the acute and post-acute post-trauma time windows may be protective of developing PTSD altogether and represents an important area for the development of early, preventative interventions.

Overall, emotional support was also associated with lower PCL-5 scores across all symptom criteria (B-E). Primarily, we found that emotional support had the highest magnitude of correlation with cluster D symptoms. Unsurprisingly emotional support can prevent social isolation in interpersonal relationships and protect against initial cognitive changes. These networks can promote recovery. Among veterans, higher levels of pre-treatment emotional support were associated with a greater reduction in PTSD symptoms following treatment with exposure therapy, and that successful psychotherapy contributed to subsequently maintained perceptions of social support [38], [39]. Similarly, civilians with chronic PTSD who were able to process their traumatic experiences with significant others, also demonstrated better treatment outcomes in both exposure therapy and cognitive restructuring [40]., Both studies demonstrate how the presence of emotionally and socially supportive relationships can improve cognitive reappraisal, mitigate stress responses, and promote coping strategies that survivors can implement when facing subsequently threatening stimuli.

Emotional support was also correlated with lower cluster E symptoms. A study by Schell et al., demonstrated that increased hyperarousal contributed to the severity of PTSD scores compared to other criteria and were also associated with a lower reduction of PCL-5 scores at a year post-trauma [41]. While we often consider irritability from an internalized perspective, it is important to recognize that there is a shared burden in trauma recovery between survivors and support networks, and it can be comparably difficult for these support networks to engage in emotionally burdensome relationships.

Conversely, we observe an almost mirrored effect from PTSD symptom severity on perceived emotional support at the 6-month timepoint. Specifically, criterion C symptoms were most correlated with a reduction in subsequent social support. Avoidance is complementary to isolation, and in PTSD can be expected to undermine social and interpersonal relationships [5]. This is further concerning in considering how emotional avoidance with diminished social support has been shown to predict increased severity in depression post-trauma [31]. What results is a cyclical and compounding series of factors, beginning with emotional avoidance leading to self-isolation and ultimately contributing to worsening PTSD symptoms, which widens the barrier for survivors to form social connections with either providers who can implement cognitive behavioral therapy, or significant others who can assist with emotionally processing trauma.

### 4.1 Limitations

Key aspects of our study limit the application of our results. Despite studies on the reliability of the NIH PROMIS emotional support questionnaire, these survey measures do not encapsulate the breadth and diversity of social support as they vary on an individual and population scale. This is evident with the lack of a consistent social support scale used in current literature The types of support required by individuals can differ between physical, emotional, socioeconomic, and even cultural aspects, and scoring measures must reflect this Similarly, for the size and scope of the study, utilizing the PCL-5 scoring scale was an appropriate screen for post-traumatic stress, and suggests risk of PTSD well. However, we recognize that the clinician administered PTSD scale is the diagnostic gold standard for PTSD and could enhance future studies on the development of PTSD in recovery.

We also aimed to maximize the generalizability of our study by collaborating with emergency departments in multiple metropolitan areas across the United States, to recruit study participants of a diverse socioeconomic background. However, for our study population nearly two-thirds of traumatic events in this AURORA data subset were classified under motor vehicle collision, while sexual assault or other forms of interpersonal trauma were less represented. While this reflects the real-time proportion of cases seen in the emergency department, interpersonal traumas may have a more significant effect on the quality of social support, while a generally increased variety in criterion A trauma could influence the range of PTSD symptom scores [42], [43]. Our study focuses on results up to 12 months post-trauma, but observational data collected on a longer time frame would provide a more comprehensive perspective on the protective effects of social support and erosive nature of traumatic stress. Additionally, our statistical models and study design are limited by correlational conclusions, and further studies need to focus on causal mechanisms surrounding how social support mitigates traumatic stress.

### 4.2 Future directions

Altogether, our results demonstrate that it is imperative that more attention be placed on enhancing, repairing, and establishing a variety of social support networks through family, friends, and caregivers, as a primary treatment to PTSD [44]. While early social and emotional support offer a protective effect against post-traumatic stress, treatment providers must be aware of how increased symptom severity can also undermine fragile social networks, nullifying the protective effect of social support on longitudinal PTSD, and worsen mental health outcomes that are comorbid to PTSD. Additional research in this field should have a two-pronged focus. Generally, defining a mechanism on how social support can buffer post-traumatic stress would be invaluable in developing socially based interventions. However, when coupled with the localization of different PTSD sub-criteria to certain brain regions, targeted treatments can be generated based on an individual’s specific symptom phenotype. Ultimately it is imperative that beginning at 1-month post-trauma, both the evolution of social networks and changing PTSD symptom severity should be monitored longitudinally through recovery, as the importance of early and sustained post-trauma social support is clear. Each point on the modified PROMIS scale grades responses to questions of interpersonal support from “rarely” to “sometimes” all the way to “very often”. If a minor increase from “rarely” to “sometimes” being subjectively supported, can contribute to nearly 20-point reductions in later PTSD symptoms, customized forms of social support could have even greater impacts on recovery. This magnitude of change is striking. Interventions to enhance early posttrauma social support may represent a low-cost and low-risk early intervention for PTSD.

## Declaration of Competing Interest

The authors declare the following financial interests/personal relationships which may be considered as potential competing interests: Dr. Rauch reported serving as secretary of the Society of Biological Psychiatry; serving as a board member of Community Psychiatry and Mindpath Health; serving as a board member of National Association of Behavioral Healthcare; serving as secretary and a board member for the Anxiety and Depression Association of America; serving as a board member of the National Network of Depression Centers; receiving royalties from Oxford University Press, American Psychiatric Publishing Inc, and Springer Publishing; and receiving personal fees from the Society of Biological Psychiatry, Community Psychiatry and Mindpath Health, and National Association of Behavioral Healthcare outside the submitted work. - Given his role as a supporting author, he had no involvement in the peer review of this article and had no access to information regarding its peer review. Full responsibility for the editorial process for this article was delegated to another journal editor. - SLR If there are other authors, they declare that they have no known competing financial interests or personal relationships that could have appeared to influence the work reported in this paper.
